# Neuropsychopharmacology of Psychosis: Relation of Brain Signals, Cognition, and Chemistry

**DOI:** 10.3389/fpsyt.2014.00076

**Published:** 2014-07-01

**Authors:** André Schmidt, Stefan Borgwardt

**Affiliations:** ^1^Department of Psychiatry (UPK), University of Basel, Basel, Switzerland

**Keywords:** psychosis, psychosis high-risk state, cognition, neuroimaging, pharmacology, computational psychiatry, brain connectivity

Recent research has resumed the pivotal significance of cognitive impairments for the development of psychosis spectrum disorders, proposing a shift in focus extending from psychotic symptoms as the key hallmarks (1, 2). Cognitive deficits are of particular interest as they precede the onset of psychosis by many years in the absence of any psychotic symptom and thus providing valuable predictions about the longitudinal course (3). Recognizing cognitive disturbances as the main promoter may allow early detection of the illness and might also lead to adequate and effective treatment.

Neuroscientific brain imaging techniques have essentially helped putting the attention back on cognition. In this research topic, we aimed at emphasizing the tremendous relevance of cognitive impairments for the early stages of psychosis and their neurobiological correlates as measured with a broad variety of brain imaging modalities such as electro- and magnetoencephalography, structural, functional, and resting state magnetic resonance imaging, near-infrared spectroscopy, and proton magnetic resonance spectroscopy. The topic begins with articles emphasizing the evidence of cognitive deficits in patients with schizophrenia, first-episode psychosis, and in persons from the general population with psychosis-like experiences and whether they are mirrored in brain signals as measured by functional near-infrared spectroscopy or structural magnetic resonance imaging (4–7). Further works review the underlying neuropharmacological mechanisms of cognitive impairments by focusing on different established domains and discuss potential drug targets for cognitive enhancement treatments (8, 9). This research topic also highlights the significance of the *N*-methyl-d-aspartate receptor for the development of psychosis and how glutamatergic metabolites are related to symptoms and cognitive function in clinical samples, suggesting promising new target pathways for the treatment of psychosis (10–12). Furthermore, electrophysiological modeling strategies in animals (13) and healthy subjects (14–16) are presented, which might help to establish neurobiological markers not only for the treatment of cognitive deficits but also for the prediction of psychosis and the development of preventive treatment schemes. The largest part of this issue unifies theoretical and experimental evidence reflecting the immense potential of computational neuroscience for shedding new light on the neurophysiological mechanisms underlying psychosis in general and on the formation of specific psychopathological signs and symptoms in particular. It starts with a normative consideration of psychotic symptoms as a result of aberrant encoding of precision embedded with predictive coding framework (17). The topic ends up with several computational modeling approaches and reviews addressing the relation between neural network properties, pharmacological challenges, cognition, and genetic risk (18–22).

This issue is intended to provide a state-of-the-art cognitive perspective to consider developing psychosis and will serve as useful framework for further investigations inferring pathophysiological mechanisms of psychosis. Such sorts of analyses might help to predate the onset of psychosis in terms of abnormal brain signals and to improve and develop new therapeutic scenarios. We would like to thank all the authors and reviewers for their valuable contributions, as well as the Editorial Office for their help in the editing process.

## Conflict of Interest Statement

The authors declare that the research was conducted in the absence of any commercial or financial relationships that could be construed as a potential conflict of interest.
